# RING-Finger Protein 6 promotes Drug Resistance in Retinoblastoma *via* JAK2/STAT3 Signaling Pathway

**DOI:** 10.3389/pore.2022.1610273

**Published:** 2022-03-18

**Authors:** Yong Chai, Shoufeng Jiao, Xin Peng, Qiang Gan, Leifeng Chen, Xiaolu Hu, Liang Hao, Shouhua Zhang, Qiang Tao

**Affiliations:** ^1^ Department of Ophthalmology, The Affiliated Children’s Hospital of Nanchang University, Nanchang, China; ^2^ Department of Pharmacy, The First Affiliated Hospital of Nanchang University, Nanchang, China; ^3^ Department of General Surgery, The Affiliated Children’s Hospital of Nanchang University, Nanchang, China; ^4^ Department of General Surgery, Second Affiliated Hospital of Nanchang University, Nanchang, China

**Keywords:** biomarker, signaling pathway, drug resistance, retinoblastoma, RING-finger protein 6

## Abstract

Chemotherapy is the first-line treatment for human retinoblastoma (RB), but the occurrence of drug resistance greatly limited its efficacy in practice. RING-finger protein 6 (RNF6) is an E3 ubiquitin ligase that is aberrantly upregulated in a range of cancers and plays important roles in cancer progression. However, the role of RNF6 in RB is largely unknown. In this study, we investigated the role of RNF6 in RB drug resistance. Two carboplatin-resistant RB cells, Y-79/CR and SO-Rb50/CR, were generated based on Y-79 and SO-Rb50 cells. RT-PCR and western blot analyses showed that RNF6 expression on both mRNA and protein levels was significantly increased in Y-79/CR and SO-Rb50/CR cells comparing to their parental cells. Knockdown of RNF6 using siRNA in Y-79/CR and SO-Rb50/CR cells resulted in cells sensitive to carboplatin on a RNF6 siRNA dose dependent manner. Similarly, RNF6 overexpression in parental Y-79 and SO-Rb50 cells could help cells gain resistance to carboplatin on a RNF6 expression dependent manner. Signaling pathway analyses revealed that JAK2/STAT3 pathway was involved in the RNF6-induced carboplatin resistance in RB cells. We further revealed that RNF6 expression in both Y-79 and SO-Rb50 cells could render cells resistant to multiple anti-cancer drugs including carboplatin, vincristine and etoposide, an implication of RNF6 as a biomarker for RB drug resistance. Taken together, our study has revealed that RNF6 is upregulated in drug-resistant RB cells and RNF6 promotes drug resistance through JAK2/STAT3 signaling pathway. The importance of RNF6 in RB cells drug resistance may represent this protein as a potential biomarker and treatment target for drug resistance in RB.

## Introduction

Retinoblastoma (RB) is an intraocular tumor that originates from the photoreceptor precursor cells of the developing retina (1). RB is one of the most common malignant intraocular tumors in children, with the incidence of 1:15,000 to 1: 20,000 new births worldwide (2). Common treatment options for RB are chemotherapy and enucleation. Carboplatin, with or without the co-administration of vincristine and etoposide, is the widely used systemic chemotherapy for RB (3). Albeit chemotherapy can greatly increase the rate of survival and prognosis, long-term treatment could lead to drug resistance which adversely affects the efficacy of chemotherapy (3). Therefore, elucidation of the mechanisms underlying RB drug resistance is of great importance to provide solutions to reduce drug resistance and consequently to improve chemotherapy efficacy (4,5).

RING-finger protein 6 (RNF6) is an E3 ubiquitin ligase (6). Although it was initially believed to be a tumor suppressor, recent studies have accumulatively indicated this protein functions as an oncogene and plays important roles in tumor development (7). Recent studies have shown that RNF6 expression is upregulated in several cancers including colorectal, breast, prostate and gastric cancers (7–12). In addition, it seems that RNF6 functions distinctively in different cancers. In colorectal cancer, RNF6 upregulation promotes tumorigenesis by Wnt/β-catenin or JAK/STAT3 pathways and is associated with poor outcome (8,9). In prostate cancer, increased RNF6 expression promotes cancer development by enhancing the transcriptional activity of androgen receptor (12). However, current knowledge of RNF6 is still very limited and the role of RNF6 in RB remains unclear.

In the current study, we established two drug-resistant RB cell lines and identified that RNF6 was upregulated in drug-resistant RB cells. Moreover, RNF6 promotes RB cell surviving drug treatment through JAK2/STAT3 pathway, and overexpression of RNF6 in drug-sensitive RB cells could render the cells resistant to multiple chemotherapeutical drugs. The findings of our study indicate that RNF6 plays an important role in RB drug resistance and may serve as a biomarker and treatment target for RB drug resistance.

## Materials and Methods

### Cell Culture

Human retinoblastoma (RB) cell lines Y-79 and SO-Rb50 were obtained from the Type Culture Collection of the Chinese Academy of Sciences and the Zhongshan Ophthalmic Center of Sun Yat-sen University, respectively. Both cell lines were cultured in RPMI 1640 (Sigma-Aldrich) supplemented with 10% FBS (HyClone, Cytiva) and penicillin (50 U/ml; Sigma-Aldrich) streptomycin (50 μg/ml; Sigma-Aldrich) in a 37°C, 5% CO_2_ incubator.

### Establishment of Drug-Resistant Cells

Drug-resistant RB cells were established as previously described with modifications (3,13). In brief, Y-79 and SO-Rb50 cells were treated with slowly increasing doses of carboplatin until cells exhibited exponential growth at high drug concentration. Cells were treated with the initial carboplatin concentration of 0.05 µM, and the treatment dose was doubled every 10 days, for 100 days. The cells surviving carboplatin treatment were then cultured in drug-free medium (complete culture medium without carboplatin) for 2 weeks before downstream tests. The carboplatin-resistant Y-79 and SO-Rb50 cells were designated as Y-79/CR and SO-Rb50/CR, respectively.

### Real Time-PCR

The relative RNA level of RNF6 in RB cells was determined by RT-PCR, as previously described with modifications (7,14). In brief, total RNA was first extracted from Y-79, Y-79/CR, SO-Rb50 and SO-Rb50/CR cells using RNeasy Mini Kit (Qiagen), according to the manufacturer’s instructions. Then, extracted RNA was reverse transcribed into cDNA with TransScript® First-Strand cDNA Synthesis SuperMix (TransGen) according to the manufacturer’s instructions. The RNF6 mRNA level was then semi-quantified using a SYBR Green-based RT-PCR method on a Bio-Rad CFX Connect platform. The following primer pairs were used: RNF6, forward 5′-AGA​AGA​TGG​CAG​CAA​GAG​CG-3′ and reverse 5′-TCA​AGT​CAG​GCT​GAG​ATG​CTA​GT-3′; β-actin, forward 5′-CAC​CAA​CTG​GGA​CGA​CAT-3′ and reverse 5′-ACA​GCC​TGG​ATA​GCA​ACG-3′. The following thermal cycling protocol was used: 95°C, 1 min; 40 cycles of 95°C, 10 s and 60°C, 30 s. The relative RNF6 mRNA level was calculated with the 2^−ΔΔCt^ using β-actin was an internal control.

### Plasmid

The RNF6 expression plasmid (p-RNF6) was constructed as previously described with modifications (15). In brief, total RNA was prepared from Y-79 cells using RNeasy Mini Kit (Qiagen) and then reverse-transcribed into cDNA with TransScript® First-Strand cDNA Synthesis SuperMix (TransGen) according to the manufacturer’s instructions. The full-length RNF6 gene was then applified by PCR using the cDNA as template. The primers used for PCR were: forward 5′-CCC​GGA​ATT​CAT​GAA​TCA​GTC​TAG​ATC​GAG​ATC​AG-3′ and reverse 5′-AAA​TAT​GCG​GCC​GCT​TAC​CCA​TTG​TTT​GCT​ATG​TTA​GAC​CC-3′. The applified RNF6 gene was inserted into expression vector pcDNA3.1 between EcoRI and NotI restriction sites. The gene sequence was verfied by Sanger sequencing.

### Cell Transfection

The knockdown of RNF6 expression in Y-79/CR and SO-Rb50/CR cells was done with siRNA transfection, while the overexpression of RNF6 in Y-79 and SO-Rb50 cells was done with p-RNF6 plasmid transfection (100 ng/well was used for 96-well plates and 500 ng/well was used for 24-well plates, unless otherwise indicated). For all the transfections, cells were seeded at 1 × 10^4^/well for 96-well plates and 5 × 10^4^ for 24-well plates, 1 day before transfection. Both siRNA and plasmid were transfected using Lipofectamine 3,000 (Thermo Fisher Scientific), according to the manufacturer’s instructions. The following siRNAs (1 pmol/well was used for 96-well plates and 5 pmol/well was used for 24-well plates, unless otherwise indicated) were used in the current study: human RNF6 siRNA (HSS165268, Thermo Fisher Scientific), human p65 siRNA (sc-29410, Santa Cruz) and negative control siRNA (4404021, Thermo Fisher Scientific). The transfection complexes were removed and replaced with fresh culture medium after 6 h.

### Drug Treatment

Y-79/CR and SO-Rb50/CR cells, and their parental cells (Y-79 and SO-Rb50) were treated with either ascendent doses of carboplatin or a fixed dose (50 µM) of carboplatin, vincristine, and etoposide for 24 h, and then cell viability was assessed. If cells were transfected with siRNA or plasmid and also treated with drugs, drugs were introduced at 24–26 h after transfection for another 24 h.

### Signaling Pathway Inhibition

Signaling pathway inhibition was done using specific pathway inhibitors, as previously described with modifications (16). In brief, cells were first seeded in 24-well plates, and then specific signaling pathway inhibitors were added into the culture for 24 h. The following signaling pathway inhibitors were used in the current study: BAY-11–7,082 (10 μM; NF-κB inhibitor), CCT251545 (10 μM; Wnt inhibitor), FLLL32 (5 μM; JAK2/STAT3) inhibitor, PD98059 (20 μM; MEK inhibitor), SB203580 (10 μM; p38 inhibitor) and SP600125 (10 μM; JNK inhibitor). All the inhibitors were purchased from Selleck and used according to the manufacturer’s instructions.

### Cell Viability Assay

Cell viability was assessed with MTT assay (R&D systems), as previously described with modifications (17,18). In brief, after 24 h of drug treatment, cells were first treated with 10-fold diluted MTT reagent for 4 h, and then Detergent Reagent was added, and the plate was further incubated for another 4 h in the dark. After incubation, the plate was read on a microplate reader, with the testing wavelength of 570 nm and the reference wavelength of 650 nm. The cell viability was calculated using cells without treatment being considered as 100% viable. Drug resistance index (RI) was defined as the ratio of IC50 of the drug-resistant cells divided by the IC50 of its parental cells.

### Western Blot

Western blot was performed as previously described with modifications (18). In brief, cells were lysed with RIPA cell lysis buffer supplemented with protease inhibitors and phosphatase inhibitors (Santa Cruz). Lysed cell samples were centrifuged at 10,000 g for 10 min at 4°C, and cleared cell lysate was then mixed with loading buffer (Beyotime) and loaded onto a 10% SDS-PAGE gel. After electrophoresis, the resolved proteins were transferred from the gel onto a PDVF membrane. The membrane was subsequently blocked with 5% non-fat milk for 1 h at room temperature. After blocking, the membrane was sequentially incubated with primary antibodies and HRP-conjugated secondary antibodies over night at 4°C and 1 h at room temperature, respectively. Following incubations, the membrane was extensively washed with PBST and the immuno-bands were visualized with ECL substrate (Beyotime). Band density was analyzed using ImageJ 1.8.0(19). The following primary antibodies were used: rabbit anti-human RNF6 antibody (ab204506; Abcam), rabbit anti-human p65 antibody (10745-1-AP, Proteintech), mouse anti-human STAT3 antibody (ab119352; Abcam), rabbit anti-human p-STAT3 antibody (9,145; Cell Signalling Technology) and mouse anti-human β-actin antibody (66009-1-Ig, Proteintech). The following secondary antibodies were used: HRP-conjugated goat anti-rabbit IgG (H + L) (SA00001-2; Proteintech) and HRP-conjugated goat anti-mouse IgG (H + L) (SA00001-1; Proteintech). All primary antibodies were used at 1:1,000 dilution and all secondary antibodies were used at 1:20,000 dilution.

### Statstical Analysis

All data were expressed as mean ± standard deviation (SD) and all statistical analyses were performed with GraphPad Prism 9.2 (GraphPad). Mann-Whitney test was used for comparisons between two groups and Kruskal-Wallis test followed by Dunn’s multiple comparisons were used for comparing three or more groups. For all the tests, a *p* value less than 0.05 was considered statistically significant.

## Results

### RING-Finger Protein 6 is Upregulated in Carboplatin-Resistant Retinoblastoma Cells

**FIGURE 1:**
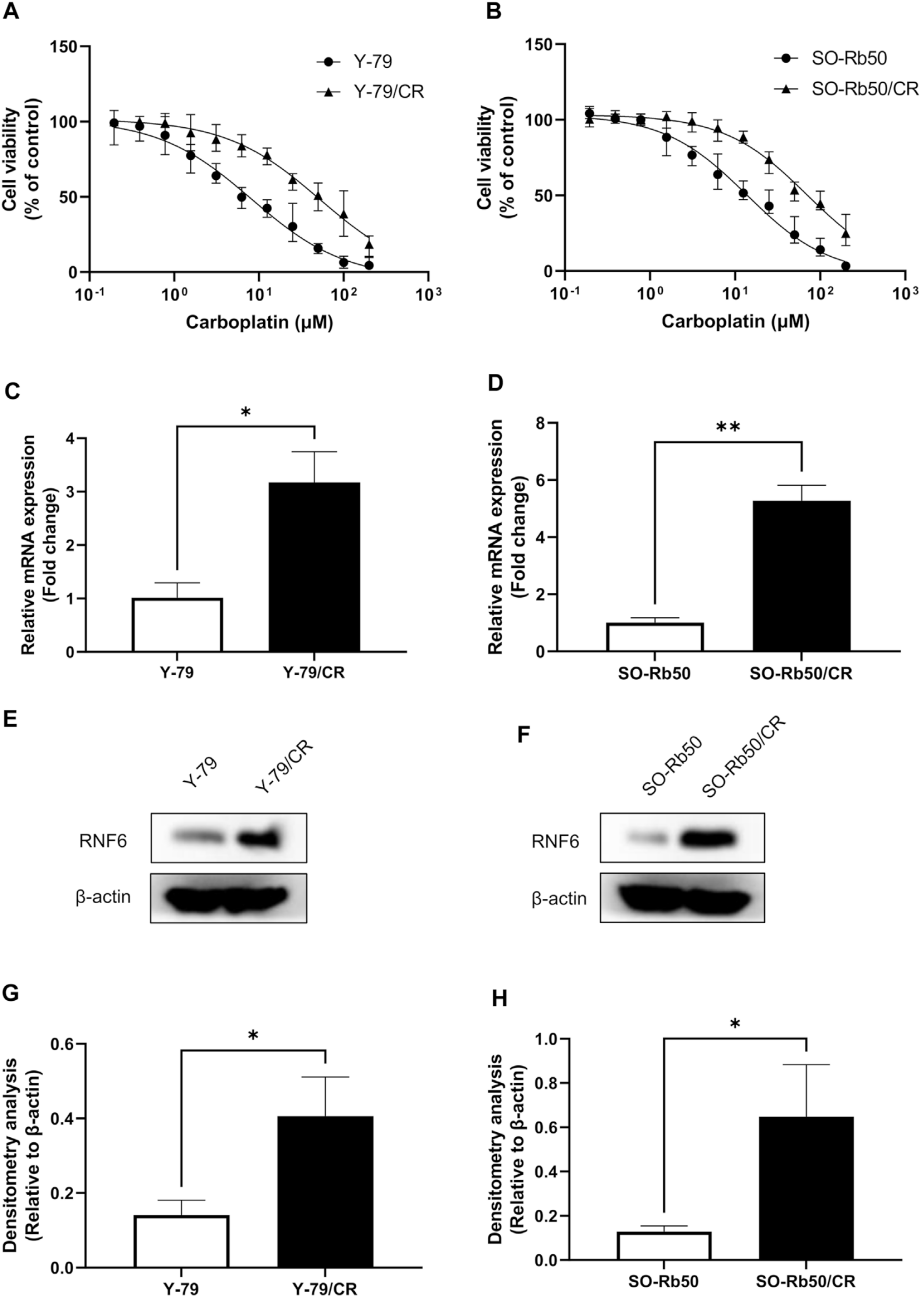
RNF6 is up-regulated in carboplatin-resistant RB cells. **(A)** Carboplatin-resistant Y-79/CR and its parental cell line Y-79 and **(B)** carboplatin-resistant SO-Rb50/CR and its parental cell line SO-Rb50 were first treated with various doses of carboplatin for 24 h, and then cell viability was assessed by MTT assay. Cells without carboplatin treatment were used as control and were considered 100% viable. Data shown are mean ± SD of three independent experiments. **(C,D)** The mRNA and **(E,F)** protein levels of RNF6 in Y-79/CR and SO-Rb50/CR and their parental cells were tested by **(C,D)** RT-PCR and **(E,F)** western blot, respectively. **(C,D)** Data shown are mean ± SD of three independent experiments. **(E,F)** One representative result is shown. **(G,H)** Densitometry analysis was performed for **(E,F)**, respectively. *, *p* < 0.05; **, *p* < 0.01.

The sensitivity of the carboplatin-resistant cells and parental RB cells to carboplatin was assessed by cell viability assay. Our data showed that carboplatin-resistant RB cells could survive much higher dose of carboplatin treatment comparing to their parental cells (Figures 1A,B). The calculation of the IC50s of carboplatin on these cells further revealed that the IC50s of carboplatin on Y-79 and Y-79/CR were 7.63 µM and 48.39 µM, respectively. This represented a resistance index (RI) of 6.34 on Y-79/CR cells (Table 1). Similarly, the IC50s of the same drug on SO-Rb50 and SO-Rb50/CR were 13.92 µM and 71.11 µM, representing a RI of 5.11 on SO-Rb50/CR (Table 1). These data here indicate that the carboplatin-resistant Y-79/CR and SO-Rb50/CR cells were successfully established.

**TABLE 1:** IC50 and resistant index of carboplatin-resistant and parental RB cells.

	IC50 (µM)	Resistant index (RI)
Y-79	7.63	—
Y-79/CR	48.39	6.34
SO-Rb50	13.92	—
SO-Rb50/CR	71.11	5.11

To test the role of RNF6 in RB drug resistance, the expression of RNF6 was then measured in Y-79/CR and SO-Rb50/CR and their parental cells. RT-PCR analysis revealed that RNF6 mRNA level was significantly increased in both Y-79/CR and SO-Rb50/CR, comparing to the parental cell line Y-79 and SO-Rb50, respectively (Figures 1C,D). In accordance, our western blot data showed that the RNF6 protein level was also considerably increased in the carboplatin-resistant RB cells comparing to the parental cells (Figures 1E,F). Together, our data here indicate that RNF6 is upregulated in carboplatin-resistant RB cells.

### RING-Finger Protein 6 Knock-Down Increases Carboplatin Sensitivity in Drug-Resistant Retinoblastoma Cells

**FIGURE 2:**
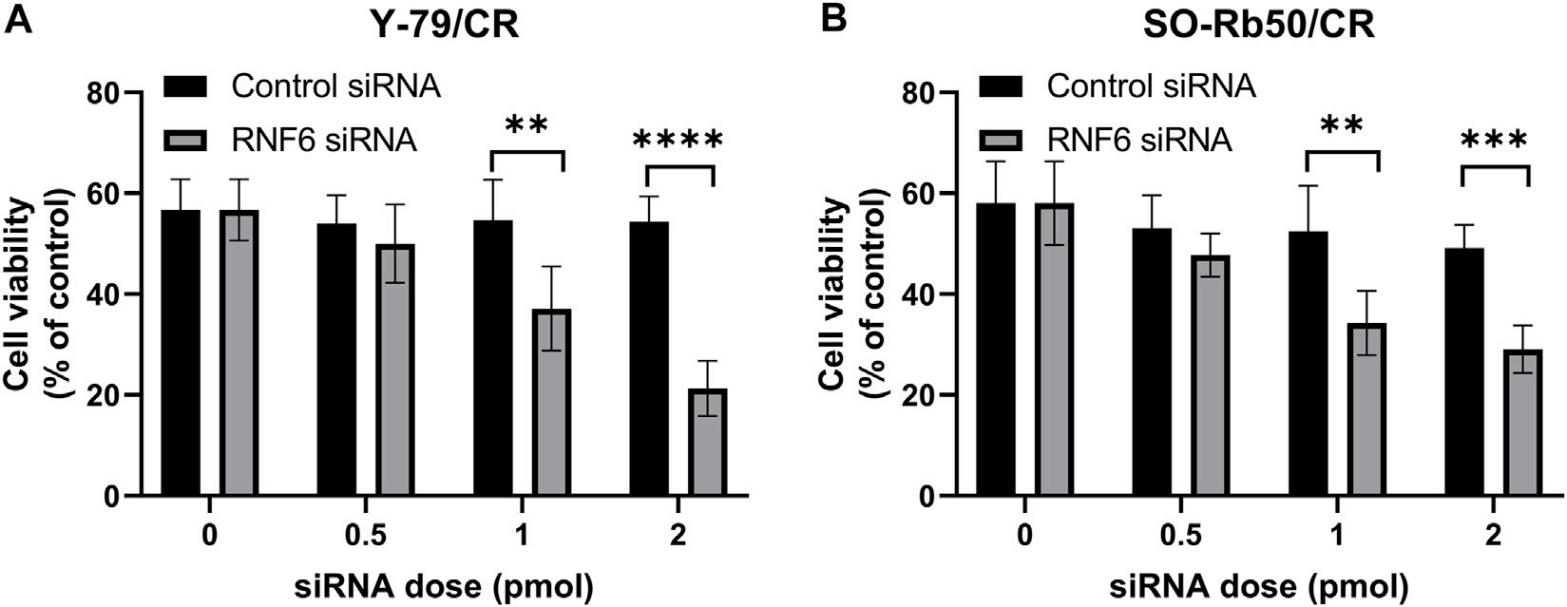
RNF6 knock-down increases carboplatin sensitivity in carboplatin-resistant retinoblastoma cells. Carboplatin-resistant RB cells **(A)** Y-79/CR and **(B)** SO-Rb50/CR were first transfected with control siRNA or RNF6 siRNA, and then cells were treated with carboplatin for 24 h. After treatment, cell viability was assessed by MTT assay. Cells with neither siRNA transfection nor carboplatin treatment were used as control and were considered 100% viable. Data shown are mean ±SD of three independent experiments. **, *p* < 0.01; ***, *p* < 0.001; ****, *p* < 0.0001.

To further investigate the importance of RNF6 in RB drug resistance, RNF6 was knocked down with specific siRNA in carboplatin-resistant Y-79/CR and SO-Rb50/CR cells, and then the cell sensitivity to carboplatin was assessed. As shown in Figures 2A,B, the knock-down of RNF6 in both Y-79/CR and SO-Rb50/CR cells rendered the cells more sensitive to carboplatin, and the cell sensitivity to the drug appeared to be RNF6 siRNA dose dependent. The data implies that RNF6 expression might enhance carboplatin resistance in RB cells.

### RING-Finger Protein 6 Overexpression Promotes Carboplatin Resistance in Retinoblastoma Cells

**FIGURE 3:**
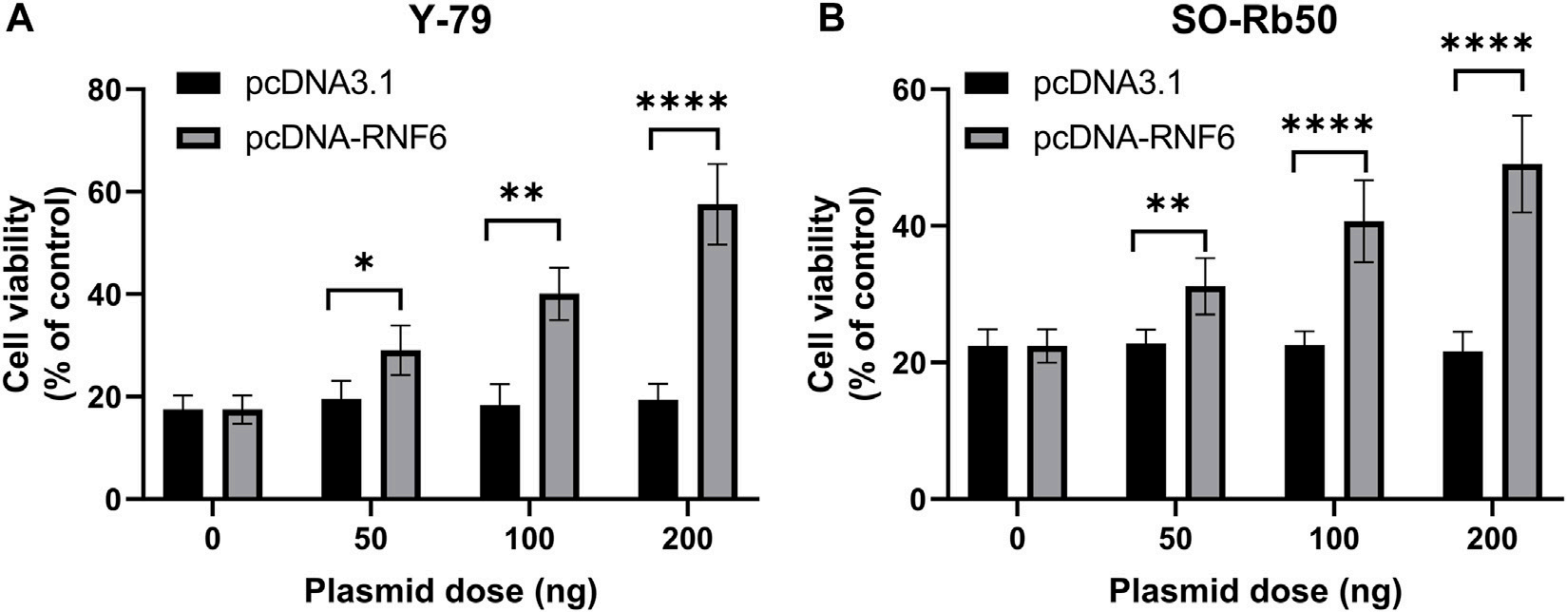
RNF6 overexpression promotes carboplatin resistance in retinoblastoma cells. RB cells **(A)** Y-79 and **(B)** SO-Rb50 were first transfected with control plasmid pcDNA3.1 or RNF6-coding plasmid pcDNA-RN6, and then cells were treated with carboplatin for 24 h. After treatment, cell viability was assessed by MTT assay. Cells with neither plasmid transfection nor carboplatin treatment were used as control and were considered 100% viable. Data shown are mean ±SD of three independent experiments. *, *p* < 0.05; **, *p* < 0.01; ***, *p* < 0.001; ****, *p* < 0.0001.

To further confirm that RNF6 expression was associated with RB drug resistance, RNF6 overexpression was performed in drug-sensitive Y-79 and SO-Rb50 cells, and then cell sensitivity to carboplatin was assessed. Our data showed that RNF6 overexpression significantly enhanced cell tolerance to carboplatin in both Y-79 and SO-Rb50 cells and such enhanced drug tolerance increased with p-RNF6 plasmid dose (Figures 3A,B). Taken together, these data further confirmed that RNF6 promotes carboplatin resistance in RB cells, implying that RNF6 may serve as a biomarker for RB carboplatin resistance.

### RING-Finger Protein 6 Promotes Carboplatin Resistance Through JAK2/STAT3 Signaling Pathway

**FIGURE 4:**
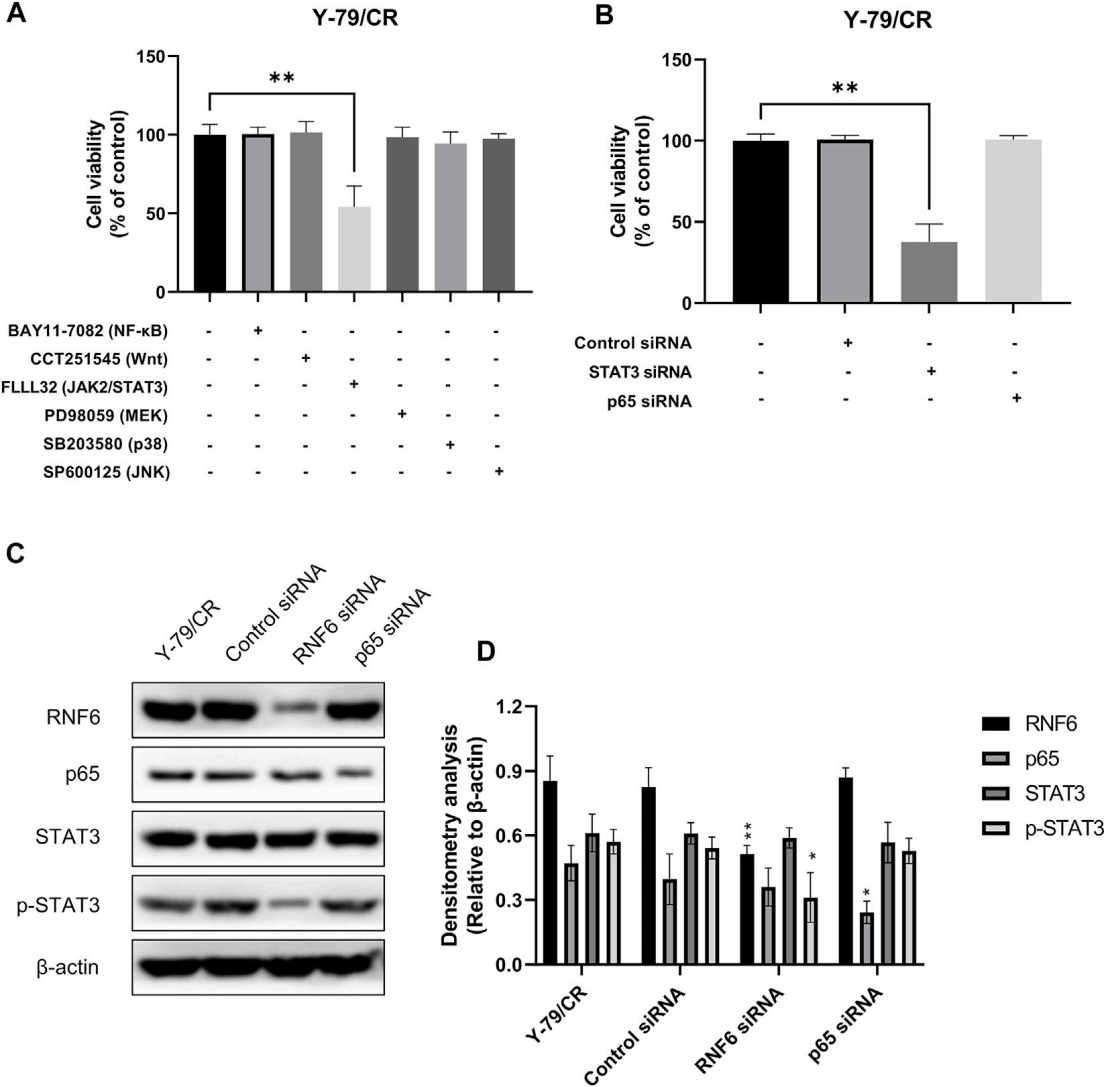
RNF6 promotes carboplatin resistance through JAK2/STAT3 signaling pathway. **(A)** Carboplatin-resistant RB cells Y-79/CR were first treated with carboplatin in the presence or absence of various signaling pathway inhibitors for 24 h, and then cell viability was assessed by MTT assay. Cells without signaling pathway inhibitor treatment were used as control and were considered 100% viable. Data shown are mean ±SD of three independent experiments. **, *p* < 0.01. **(B)** Carboplatin-resistant RB cells Y-79/CR were first transfected with control siRNA, STAT3 siRNA or NF-κB siRNA, and then treated with carboplatin for 24 h. After treatment, cell viability was assessed by MTT assay. Cells without siRNA transfection were used as control and were considered 100% viable. Data shown are mean ±SD of three independent experiments. **, *p* < 0.01. **(C)** Carboplatin-resistant RB cells Y-79/CR were first transfected with control siRNA, STAT3 siRNA or NF-κB siRNA, and then treated with carboplatin for 24 h. After treatment, the expression of RNF6, p65, STAT3 and p-STAT3 was determined by western blot. One representative result is shown. **(D)** Densitometry analysis was performed for **(C)**. *, *p* < 0.05; **, *p* < 0.01.

We next further explored the possible mechanism underlying RNF6-associated carboplatin resistance in RB cells. In this experiment, Y-79/CR was used a carboplatin-resistant RB cell model. To check whether RNF6 induced carboplatin resistance in RB cells through certain signaling pathways, Y-79/CR cells were first treated with a panel of specific inhibitors targeting common drug resistance pathways and then the cell sensitivity to carboplatin was assessed. As shown in Figure 4A, of the tested 6 pathway inhibitors, only FLLL32, a specific inhibitor for JAK2/STAT3 pathway, significantly decreased the tolerance of Y-79/CR cells to carboplatin treatment, while the other 5 inhibitors showed no apparent impact on cell survival. To further confirm the involvement of JAK2/STAT3 pathway in carboplatin resistance in RB cells, Y-79/CR cells were first transfected with or without STAT3 siRNA or NF-κB siRNA, and then cell sensitivity to carboplatin was determined. As shown in Figure 4B, knock-down of STAT3 with siRNA dramatically decreased the cell viability upon carboplatin treatment, while NF-κB knock-down or control siRNA transfection did not induce apparent change in cell viability. These data together indicate that JAK2/STAT3 pathway is involved in carboplatin resistance in RB cells.

The involvement of JAK2/STAT3 pathway in RNF6-induced carboplatin resistance in RB cells was also investigated. Y-79/CR cells were first mock-treated, or treated with control siRNA, RNF6 or p65 siRNA, and the expression of p65, STAT3 and p-STAT3 was determined by western blot. Our data showed that RNF6 knock-down with RNF6 siRNA did not change the expression of STAT3, but inhibited the phosphorylation of this protein, as evidenced by the decrease of p-STAT3 (Figure 4C). By contrast, p65 knock-down did not show impact on either STAT3 or p-STAT3. These data indicate that RNF6 promotes carboplatin resistance in RB cells through JAK2/STAT3 pathway.

### RING-Finger Protein 6 Promotes Multidrug Resistance in Retinoblastoma Cells

**FIGURE 5:**
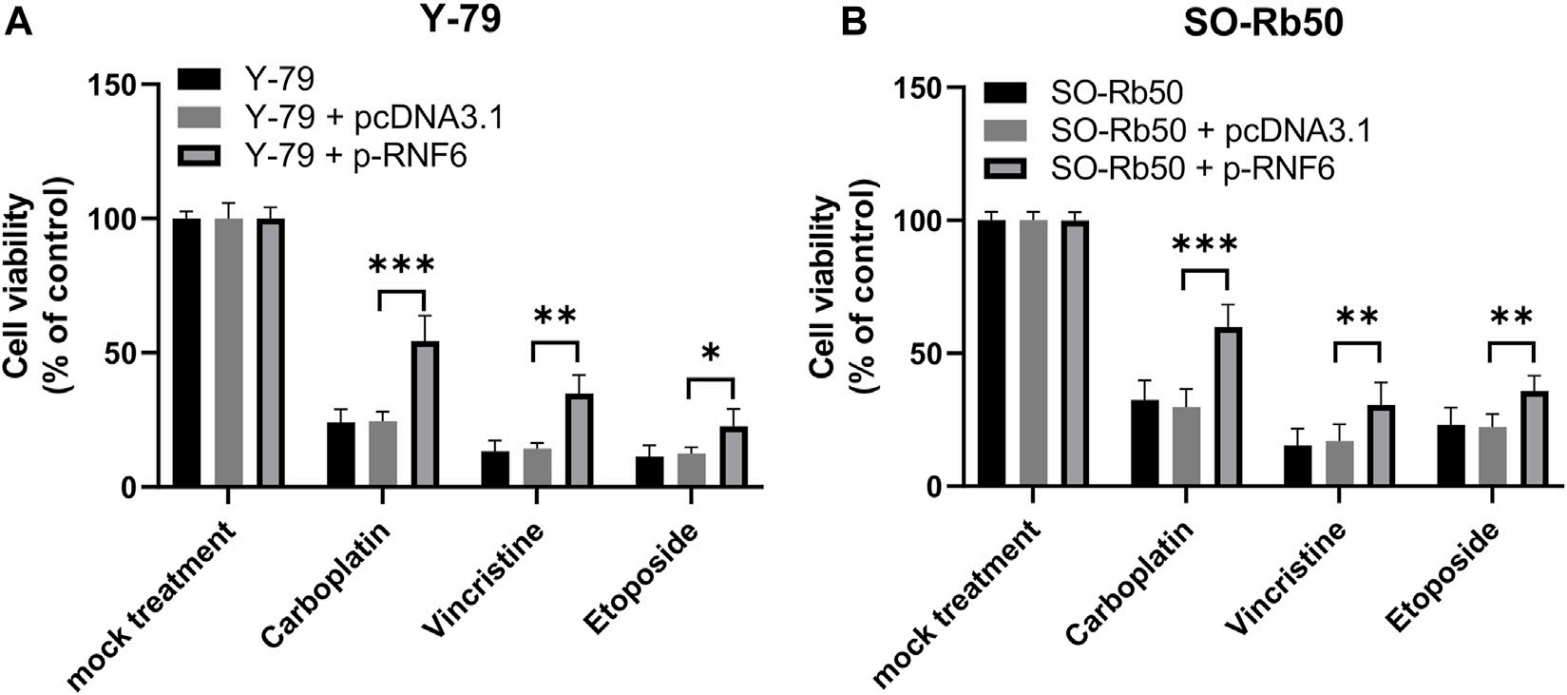
RNF6 expression promotes multidrug resistance in retinoblastoma cells. RB cells **(A)** Y-79 and **(B)** SO-Rb50 were first transfected with control plasmid pcDNA3.1 or RNF6-coding plasmid pcDNA-RN6, and then cells were treated with carboplatin, vincristine and etoposide for 24 h. After treatment, cell viability was assessed by MTT assay. Cells without plasmid transfection and drug treatment were used as control and were considered 100% viable. Data shown are mean ±SD of three independent experiments. *, *p* < 0.05; **, *p* < 0.01; ***, *p* < 0.001.

To test if RNF6 upregulation could render RB cells resistant to anti-cancer drugs besides carboplatin, Y-79 and SO-Rb50 cells were first overexpressed with RNF6 and then their sensitivities to carboplatin, vincristine and etoposide were assessed. Similar to the data obtained from carboplatin, these two RB cell lines with RNF6 overexpression demonstrated significantly increased tolerance to these drugs (Figures 5A,B). These data indicate that RNF6 can promote RB cells resistant to multiple anti-cancer drugs and may serve as a biomarker and treatment target for drug resistance in RB cells.

## Discussion

Drug resistance remains a major constraint in RB chemotherapy treatment, leading to treatment failure or patient relapse. Understanding the mechanisms underlying cancer drug resistance is essential for the development of novel therapeutic strategies to overcome such problems and enhance efficacy in RB treatment. Our current study showed that RNF6, an E3 ubiquitin ligase, was upregulated in drug-resistant RB cells, and RNF6 promoted drug resistance through JAK2/STAT3 signaling pathway. Furthermore, overexpression of RNF6 in sensitive RB cells could render cells resistant to multiple chemotherapeutic drugs. The findings of our study have highlighted the importance of RNF6 in RB drug resistance, which not only provides a potential target for drug resistance treatment, but also a potential biomarker for diagnosis.

Biomarkers are useful for disease diagnosis and may also serve as prognostic indicators. In RB, many biomarkers for diagnosis and prognosis have been identified and studied (20). However, few studies have been done for the identification of biomarkers for RB drug resistance. As drug resistance slowly occur throughout the chemotherapy treatment course, the expression of a variety of related genes may be altered in cancer cells, leading to drug resistance (21). FoxM1, a member of the Forkhead transcription factor family, is upregulated in carboplatin-resistant Y-79/CR cells (3). In another study, several genes including ARHGAP9, HIST1H4H, RELN, DDIT4, HK2, STC1 and PFKFB4 were elevated on mRNA level in etoposide-resistant Y-79/EDR cells (22). Albeit these genes/proteins may serve as biomarkers for drug resistance in RB, they have not been tested if their upregulation is universal in all RB cells and all chemotherapy drugs. In our study, we have shown that RNF6 is upregulated in both Y-79/CR and SO-Rb50/CR cells, and more importantly, upregulation of RNF6 in drug-sensitive RB cells Y-79 and SO-Rb50 renders cells resistant to various chemo-drugs. Although further investigations are needed in the clinical settings, RNF6 has the potential to be a biomarker for drug resistance in RB.

RNF6 is an E3 ubiquitin ligase and increasing evidence has indicated that RNF6 is involved in the development of various cancers (7,8,12,23,24). As a ubiquitin ligase, RNF6 regulates numerous cellular processes by ubiquitin-dependent degradation of target proteins (25). Based on the current findings, RNF6 seems to promote carcinogenesis by targeting different proteins in different cancers. In colorectal cancer, RNF6 ubiquitinates and degrades the transcriptional TLE3 through Wnt/β-catenin pathway (8). In gastric cancer, RNF6 regulates cancer cell growth by affecting the SHP-1/STAT3 signaling pathway (7). Whereas in myeloid leukaemia, RNF6 promotes tumor growth by activating the PI3K/AKT/mTOR signaling pathway (15,24). The involvement of RNF6 in chemo-resistance remains largely unknown. In our current study, we have identified that RNF6 can promote drug resistance in RB through JAK2/STAT3 signaling pathway. Although beyond the scope of our current study, it would be interesting to further investigate if RNF6 plays a role in the drug resistance of other types of cancers. Given the importance of RNF6 in RB drug resistance, it would also be valuable to check if RNF6 could be a therapeutic target for treating drug resistance in RB and/or other cancers. In addition, RB is usually initiated because of biallelic loss of tumor suppressor gene *RB1*, and whether there is a causal relationship between the loss of RB1 function and RNF6 upregulation in RB remains to be elucidated (26).

STAT3, the signal transducer and activator of transcription 3, is an oncogenic transcription factor and its role in tumorigenesis has been widely studied (27). STAT3 activation promotes cancer cell growth and metastasis and also induces chemoresistance in many cancers (27–32). Targeting STAT3 has been considered as an ideal strategy in the treatment of various cancers (33,34). In this study, we have identified that STAT3 pathway is involved in the RNF6-induced RB drug resistance. Albeit beyond the scope of our current study, it would be warranted for future studies to investigate if targeting STAT3 can be an effective treatment for RB drug resistance.

Taken together, our current study has revealed the role of RNF6 in RB drug resistance and underlying mechanism. The findings of our study not only provide a potential treatment target for RB drug resistance, but also present a biomarker candidate for the diagnosis of drug resistance in RB.

## Conclusion

RNF6 is upregulated in carboplatin-resistant RB cells on both mRNA and protein levels, and RNF6 level is associated with the level of RB cell tolerance to carboplatin. RNF6 promotes drug resistance through JAK2/STAT3 signaling pathway and RNF6 upregulation can render cells resistant to multiple anti-cancer drugs. The findings of our study have revealed the role of RNF6 in drug resistance in RB cells and underlying mechanism. The importance of RNF6 in drug resistance in RB implies that RNF6 may serve as a biomarker and treatment target for RB drug resistance.

## Data Availability

The original contributions presented in the study are included in the article/Supplementary Material, further inquiries can be directed to the corresponding authors.
